# Domains and Methods of Medical Device Technology Evaluation: A Systematic Review

**DOI:** 10.3389/phrs.2024.1606343

**Published:** 2024-07-24

**Authors:** Fotini Santos Toscas, Daiana Laurenci Orth Blas, Leidy Anne Alves Teixeira, Marisa da Silva Santos, Eduardo Mario Dias

**Affiliations:** ^1^ Technology Center for SUS/SP, Institute of Health (CTS-IS), and Radiology Program, Faculty of Medicine, State University of São Paulo (USP), São Paulo, SP, Brazil; ^2^ Health Technology Assessment Center, Faculty of Technology of Sorocaba (Fatec-SO), Sorocaba, SP, Brazil; ^3^ Brazil National Health Surveillance Agency (Anvisa), Brasília, Brazil; ^4^ Núcleo de Avaliação de Tecnologias em Saúde, National Institute of Cardiology (NATS-INC), Rio de Janeiro, RJ, Brazil; ^5^ Polytechnic School, University of São Paulo (USP), São Paulo, SP, Brazil

**Keywords:** medical devices, health technology assessment, early dialogue, coverage, real-world evidence, review, biomedical

## Abstract

**Objectives:**

Identify, through a systematic review, the main domains and methods to support health technology assessment of Medical Devices (MD) from the perspective of technological incorporation into healthcare systems.

**Methods:**

Performed structured searches in MEDLINE, Embase, BVS, Cochrane Library, and Web of Science for full studies published between 2017 and May 2023. Selection, extraction, and quality assessment were performed by two blinded reviewers, and discrepancies were resolved by a third reviewer.

**Results:**

A total of 5,790 studies were retrieved, of which 41 were included. We grouped the identified criteria into eight domains for the evaluations.

**Conclusion:**

Overall, studies discuss the need to establish specific methods for conducting HTA in MD. Due to the wide diversity of MD types, a single methodological guideline may not encompass all the specificities and intrinsic characteristics of the plurality of MD. Studies suggest using clustering criteria through technological characterization as a strategy to make the process as standardized as possible.

## Introduction

Medical devices (MD) are crucial healthcare technologies for the prevention, diagnosis, rehabilitation, and treatment of diseases, as well as in patient monitoring [1]. Recognizing the important role of these technologies in healthcare services, the World Health Assembly (WHA) adopted a resolution, WHA60.29 in May 2007, addressing the need to establish the management of these technologies [2]. In 2011, the World Health Organization (WHO) published a series of technical documents, including the thematic area of MD technology assessment, containing: i) assessment of MD needs, ii) MD procurement, iii) donations of MD, iv) MD inventory management, and v) MDmaintenance [3]. In 2014, the WHO member countries, through resolutionWHA67.23, recognized the enormous challenges in managing these technologies, particularly in low- and middle-income countries. The WHA67.23 addressed the need to establish tools to manage the use of healthcare technologies, as well as strengthen the link between technology assessment, regulation, and healthcare management [4].

In scenarios where economic resources are often scarce, the rigorous decision process for incorporating and disseminating these technologies represents a challenge for health systems. Health Technology Assessment (HTA) emerges as a process that seeks to verify whether a given technology is effective, safe and economically advantageous when compared to available alternatives. Since its inception, the field of HTA has been systematically developed by the U.S. Office of Technology Assessment, which published its first report on HTA in 1976 [5].

The HTA multidisciplinary process uses explicit methods to determine the value of a health technology at different points in its lifecycle. The HTA purpose is to inform decision-making aiming to promote an equitable, efficient, and high-quality health system [6].

The final outcome of HTA studies may vary depending on the country, the perspective adopted, the stakeholders involved and the decision-making context. The incorporation of new technologies without going through the necessary and critical HTA process can pose risks to the population and compromise the sustainability of the health system [7].

In general, HTA systematically examine various combinations of the following domains: technical performance, safety, clinical efficacy and effectiveness, cost, cost-effectiveness, organizational implications, social consequences, and legal and ethical implications of applying a health technology [8]. Choosing different domains for an assessment report depends on the specific context of the policy issue; one can focus on safety, clinical efficacy and effectiveness, and cost, such as in the pre-marketing phase, post-marketing phase, and even withdrawal of the product [7, 8].

In the specific case of medical devices, HTA has emerged as an important tool for supporting the core functions of effective and sustainable health systems and defining prioritization, incorporation and selection of MD [8]. However, unlike other health technologies, such as medicines, HTA processes in MD present gaps and weaknesses arising from the particularities and specificities of the MD sector. MD have challenged the HTA structures due to their constant technological evolution, strong dynamism of incremental innovation, learning curve, context dependence and wide heterogeneity.

There is a recognized lack of methods and tools to support HTA for MD, especially in the pre-commercialization phases. In the HTA process, there is also a lack of emphasis on organizational impact, equity, ethical issues, feasibility considerations, and acceptability by patients and healthcare providers [8].

A variety of aspects must be examined in the HTA process with a view to the implementation and adoption of these technologies in healthcare services. For highly complex MD, it is extremely important to consider the critical interdependence of these technologies. The best results cannot be achieved by investing only in a set of technologies. MD for diagnostic use must be combined with therapeutic capacity to have a significant impact on clinical management. There is a considerable risk that MD will not be used because the technology does not match the settings and resources available at the place of use [9].

The WHO technical series document “Health technology assessment of medical devices,” published in 2011 presents a set of essential elements for successful implementation of HTA projects at the national level, namely: good governance, adequate financing, adequate and good collaboration with partners. It emphasizes that health systems are strengthened when a HTA is integrated with human and material resources, in databases and sources, in decision-making, in the development of transparent policies, and linked to the global vision of equity and accountability [10].

In 2015, the WHO published the report “Developing an approach for using health technology assessments in medical product reimbursement systems,” which discussed the use of HTA in low- and middle-income countries (LMICs), including aspects of its use in relation to clinical practice, pricing policies, and reimbursement decisions. The document highlights that the use of HTA is adequately linked to other policy tools for efficient resource management, especially with regard to pricing and reimbursement policies [11].

Therefore, this review aims to characterize the domains and identify innovative methods for HTA studies with a focus on incorporating MD into healthcare systems.

## Methods

### Search Strategy and Selection Criteria

This is a systematic review following the Brazilian guideline for systematic reviews, and the Preferred Reporting Items for Systematic Reviews and Meta-Analyses (PRISMA) hecklist. All parameters were pre-defined and registered in a protocol published on the OSF platform [12]. The project was registered and is available at [13].

The search was conducted in three stages. The first stage was an initial search limited to Embase and Web of Science online databases. This initial search aimed to analyze concepts included in index terms, author’s text keywords, title and abstract of the retrieved articles, as well as to help the research question elaboration.

The research question was organized using the PICO strategy (population, intervention, control, and outcomes): What are the main methods and domains that integrate HTA studies of MD from the perspective of technological incorporation in healthcare systems?

As a criterion for the research question, we used the definition adopted by the WHO for MD: “medical devices can be any instrument, apparatus, implement, machine, appliance, implant, reagent for *in vitro* use, software, material or other similar or related article, intended by the manufacturer to be used, alone or in combination for a medical purpose” [8]. We included studies in the review if they met the eligibility criteria described in Table 1.

**TABLE 1:** Eligibility criteria (Domains and methods of medical device technology evaluation: a systematic review. São Paulo, Brazil, 2024).

Inclusion criteria	Exclusion criteria
P: HTA agencies, national health services, policymakers. Studies that provide in-depth description of the HTA process for the incorporation of MD. I: Full studies that provide in-depth description of the HTA process for the incorporation, coverage or reimbursement of MD. With a focus on capturing recent HTA processes, were included studies conducted between 2017 and May 2023 C: with or without comparator O: HTA methods and domains to support incorporation of medical devices into healthcare systems, without restrictions on the type of study design	P: regulatory agencies for registries, commercialization, surveillance I: Studies that did not explicitly demonstrate methods to support decision-making for the incorporation of MD into healthcare systems were excluded Studies with a specific clinical framework (specific disease) or specific clinical profile (pediatric, adult) were excluded. Studies with a specific level of care, such as specific application in primary care or hospital-based HTA (mini-HTA) focused on the acquisition of MD, were excluded Conference abstracts were not included; only completed and full-length articles were considered

In addition to the criteria listed above, the studies were limited to publications in English, Spanish, or Portuguese languages. There were no restrictions on the study design type.

Based on the strategy PICO question above, the second search was conducted on 14 October 2022. Then, a targeted search strategy was performed at PubMed, Embase, Virtual Health Library (BVS), Cochrane Library, Web of Science databases, using controlled vocabulary and previously identified free terms through a targeted search strategy for each database. Additionally, gray literature studies were searched from the INAHTA and the WHO. On 3 May 2023, the search was updated. The search terms used included a comprehensive combination of terms related to medical devices and HTA, such as: (“Medical Technology” OR “Medical Device” OR “Equipment and Supplies”) AND (“Health Technology Assessment” OR “Biomedical Technology Assessments”). The search strategy by specific database, its execution date and the results, can be found in Supplementary Material S1.

### Data Analysis

For the data selection processes, the online review management tool Rayyan was used. Two blinded authors (FT and DB) retrieved titles and abstracts (first-level) to establish the records suitability according to the inclusion criteria. Eligible articles were retained for the full-text article review. Reviewers’ discrepancies or disagreements were resolved by a third reviewer (LT).

For final review inclusion, once again the authors (FT and DB) independently reviewed the full-text articles. After completing the eligibility process, a consensus was established by the three authors.

The reference list of reports and articles identified as additional text studies sources was completed through the search of the INAHTA and WHO databases, using the free term “Medical Devices” and the same publication period, the last 5 years, in English, Portuguese, and Spanish languages.

The included articles and documents were then incorporated into the research management software Mendeley. Data from the studies included in the full-text were independently extracted by two authors using a standardized extraction form developed for the study in Microsoft Office Excel, according to the variables described below: Year, Title, Author, Journal, Country, Study Type, attribute, HTA Domains, HTA Method, Results, Knowledge Gaps, and Limitations. A third reviewer reconciled the collected data, and their categorization was done by consensus.

In the context of this systematic review of medical devices, the term “domain” was inspired by the EUnetHTA Core Model^®^, which are: current use, technical, safety, clinical effectiveness, cost and economic evaluation, ethical analysis, organizational, patient & social, and legal aspects. The term “domain” here can be understood as the term to designate groupings of similar characteristics and specificities of MD. Domains can be composed of dimensions (subdomains) highlighted by their relevance in the context of MD HTA. Thus, during the complete reading of the selected articles, we sought to identify which of these domains were present, which others could be added, in addition to identifying the methods used in MD HTA analyses.

Dimension percentages were calculated considering the number of times they were referenced in relation to the total number of articles selected. Similar calculations were carried out for the methods table, considering in this case the method/tools/type of analysis identified in relation to the total number of articles selected.

The assessment of methodological quality and evidence confidence was performed by peers using specific tools according to the designs of the included studies. For articles where the authors themselves did not explicitly state the category of their study, we define the classification based on information from the methods used. Supplementary Material S2 presents the concept of each type of study that we used to define the typology of included articles. Systematic review studies were evaluated using the AMSTAR 2 tool. Narrative review studies using the SANRA tool. In cases where the methodology was similar to the experience report, or descriptive review or research survey were evaluated using the JBI Text and Opinion tool. The evaluation documents, containing the summary of the methodological approach and the classification of the type of study that we defined, are available in Supplementary Material S3 and were all considered to be of good quality.

## Results

**FIGURE 1:**
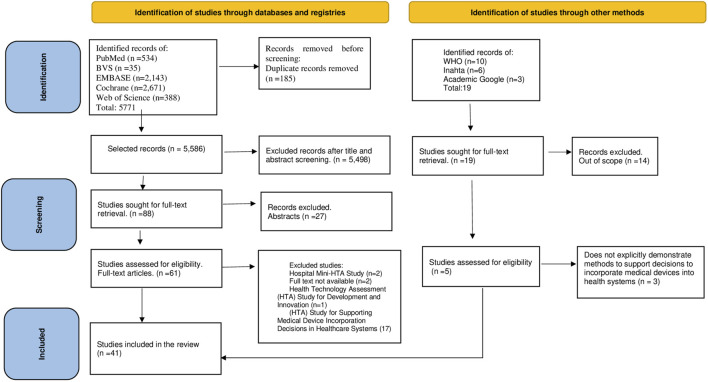
Flow chart depicting literature search and exclusion process (modified according to Preferred Reporting Items for Systematic Reviews and Meta-Analyses) (Domains and methods of medical device technology evaluation: a systematic review. São Paulo, Brazil, 2024).

The searches in the databases resulted in a total of 5,771 records. After removing duplicates, titles and abstracts of 5,586 records were analyzed, of which 88 full papers were assessed according to the eligibility criteria. Additionally, 10 publications from WHO, 6 from INAHTA, and 3 from Google Scholar were identified, of which 2 publications met the eligibility criteria. A total of 41 studies were included in this review. Figure 1 presents the PRISMA flowchart of the search and study selection process. The list of 25 studies excluded during full-text reading, along with the reasons for exclusion, is available in Supplementary Material S4.

The characteristics of the included studies are presented in Table 2. A significant number of the included studies were conducted in European countries (17/41), particularly related to the MedtecHTA Project (4/41), a specific European project on HTA for MD. Regarding the study type, the majority were reviews (35/41), including 18 descriptive reviews of HTA agency experiences, 14 narrative reviews, and three systematic reviews. There were also five survey research studies using stakeholders interview method and one WHO guideline.

**TABLE 2:** Characteristics of the included studies (Domains and methods of medical device technology evaluation: a systematic review. São Paulo, Brazil, 2024).

No	Article	Authors	Year	Country/Region	Type of study
1	A new health technology assessment system for devices: The first 5 years	Campbell et al.	2017	England	Descriptive review -
2	A review of implementation frameworks to operationalize health technology assessment recommendations for medical technologies in the Singapore setting	Segar et al	2021	Republic of Singapore	Descriptive review - experience
3	Analysis of duplication and timing of health technology assessments on medical devices in Europe	Hawlik et al	2018	Europe	Descriptive review - cohort study
4	Assessing the value of innovative medical devices and diagnostics: the importance of clear and relevant claims of benefit	Campbell et al	2018	Europe	Descriptive review - experience
5	Assessment of Devices, Diagnostics and Digital Technologies: A Review of NICE Medical Technologies Guidance	Crispi	2019	Europe	Descriptive review - experience
6	Challenges in the Assessment of Medical Devices: The MedtecHTA Project	Tarricone et al	2017	Europe	Narrative review
7	Challenges of Health Technology Assessment in Pluralistic Healthcare Systems: An ISPOR Council Report	Drummond et al	2022	N/A	Narrative review
8	Challenges with coverage with evidence development schemes for medical devices: A systematic review	Reckers-Droog	2020	Europe	Systematic review
9	Characterising Uncertainty in the Assessment of Medical Devices and Determining Future Research Needs	Rothery	2017	N/A	Narrative review
10	Coverage with evidence development schemes for medical devices in Europe: characteristics and challenges	Federici	2021	Europe	Descriptive review - experience
11	Critical Review of European Health- Economic Guidelines for the Health Technology Assessment of Medical Devices	Blüher et al	2019	Europe	Systematic review
12	Decommissioning medical devices	WHO	2019	N/A	Guideline
13	Economic evaluation as a tool in emerging technology assessment	Bogavac-Stanojević and Nataša	2019	N/A	Narrative review
14	Eleven years of economic evaluations of medical devices by the Spanish Network of Assessment Agencies. Methodological quality and cost-utility impact	Giménez et al	2020	Spain	Descriptive review - experience
15	Eliciting preferences for medical devices in South Korea: A discrete choice experiment	Lee and Bae	2017	South Korea	Descriptive review - experience
16	Establishing a national HTA program for medical devices in Italy: Overhauling a fragmented system to ensure value and equal access to new medical technologies	Tarricone et al	2021	Italy	Descriptive review - experience
17	European Collaboration in Health Technology Assessment (HTA): goals, methods and outcomes with specific focus on medical devices	Erdös et al	2019	Europe	Descriptive review - experience
18	Exploration and preferential ranking of patient benefits of medical devices: A new and generic instrument for health economic assessments	Lesén et al	2017	Sweden	Survey research
19	Formal Implementation of Cost- Effectiveness Evaluations in Japan: A Unique Health Technology Assessment System	Hasegawa	2020	Japan	Descriptive review - experience
20	Health technology assessment methods guidelines for medical devices: How can we address the gaps? The International Federation of medical and Biological Engineering perspective	Polisena et al	2018	N/A	Narrative review
21	Health technology assessment of medical devices	Polisena et al	2019	N/A	Narrative review
22	Health technology assessment of medical devices in low- and middle-income countries: Study design and preliminary results	Pecchia	2017	N/A	Narrative review
23	Health technology assessment of medical devices: current landscape, challenges, and a way forward	Ming et al	2022	N/A	Narrative review
24	How innovation can be defined, evaluated and rewarded in health technology assessment	Rejon-Parrilla	2022	France, Italy, England, Japan e Spain	Narrative review
25	HTA of medical devices: Challenges and ideas for the future from a European perspective	Fuchs et al	2017	Europe	Survey research
26	Implementation of coverage with evidence development schemes for medical devices: A decision tool for late technology adopter countries	Kovács et al	2022	Europe	Survey research
27	Improving the Methods for the Economic Evaluation of Medical Devices	Tarricone et al	2017	Europe	Systematic review
28	Integrating the Voice of the Patient into the Medical Device Regulatory Process Using Patient Preference Information	Benz et al	2020	N/A	Narrative review
29	Intrinsic properties of medical devices: considerations for economic evaluation	Basu and Eggington	2019	N/A	Narrative review
30	Key Recommendations from the MedtecHTA Project	Tarricone et al	2017	Europe	Narrative review
31	Postlaunch evidence-generation studies for medical devices in Spain: The RedETS approach to integrate real-world evidence into decision making	Serrano- Aguilar et al	2021	Spain	Descriptive review - experience
32	Pushing the boundaries of evaluation, diffusion, and use of medical devices in Europe: Insights from the COMED project	Torbica et al	2022	Europe	Descriptive review - experience
33	Quo Vadis HTA for Medical Devices in Central and Eastern Europe? Recommendations to Address Methodological Challenges	Daubner- Bendes et al	2021	Europe	Survey research
34	Ten recommendations for assessing the comparative effectiveness of therapeutic medical devices: a targeted review and adaptation	Schnell- Inderst et al	2018	N/A	Narrative review
35	Testing a new taxonomic model for the assessment of medical devices: Is it plausible and applicable? Insights from HTA reports and interviews with HTA institutions in Europe	Fuchs et al	2019	Europe	Survey research
36	The reimbursement coverage decisions and pricing rules for medical devices in Taiwan	O'Rourke	2020	Taiwan	Descriptive review - experience
37	Mapping of database in medical devices: review and the Brazilian scenario for evaluation with real world data (RWD)	Toscas and Teixeira	2022	Brazil	Narrative review
38	Integrating organizational impacts into health technology assessment (HTA): an analysis of the content and use of existing evaluation frameworks	Pascal et al	2022	France	Descriptive review - experience
39	The added value of applying a disinvestment approach to the process of health technology assessment in Italy	Cadeddu et al	2023	Italy	Descriptive review - experience
40	An accelerated access pathway for innovative high-risk medical devices under the new European Union Medical Devices and health technology assessment regulations? Analysis and recommendations	Tarricone et al	2023	Europe	Descriptive review - experience
41	Current Medical Technology Reimbursement System in Japan	Matsumoto et al	2023	Japan	Descriptive review -experience

The results of this review were categorized into eight domains: 1) safety, 2) effectiveness, 3) health problem, current use of technology and innovation, 4) legal, 5) organization, 6) description and technical characteristics of technology, 7) costs and economic evaluation, 8) social participation (Table 3).

**TABLE 3:** Domains to be considered in Health Technology Assessment of Medical Devices (Domains and methods of medical device technology evaluation: a systematic review. São Paulo, Brazil, 2024).

Domains	Dimension	Description	No article Table 2	% citations/total articles
Safety	-	Safety seeks to ensure that health technologies are used in a way that minimizes risks to patients and optimizes desired results. For example,: improvement in clinical outcome, increase in survival rate, improvement in quality of life, significant reduction in recurrence, complications, side effects, in duration of the procedure	1, 2, 4, 8, 10, 13, 14, 16, 17, 20, 21, 24, 25, 26, 27, 31, 32	41
Effectiveness	-	Effectiveness comprises the ability of an intervention to produce desired results under defined conditions. Example: improvement in clinical outcome, increase in survival rate, improvement in quality of life, significant reduction in recurrence, complications, side effects, reduction duration of the procedure, etc.	1,2,4, 8, 10, 11, 12, 14, 15, 16, 17, 20, 21, 25, 26, 27, 31, 40	44
Health problem, current use of technology and innovation	Evidence	Evidence: data, information or results of studies and research that are used to support clinical decisions. Example: transferability, Real Word Evidence, availability of substitutes and post-market evaluation/performance monitoring	3,4,5, 11, 14, 15, 16, 17, 21, 22, 24, 26, 27, 31, 32, 33, 37	41
	Life cycle	Analysis of innovation considering the technological life cycle (recent health registration, initial diffusion, large-scale use, disincorporation)	12, 14	5
	Incremental innovation	Analysis of innovation aspects considering added therapeutic value, non-redundancy, non-accumulative technology, change of technological and clinical route, health condition and unmet clinical needs. Analysis of the degree of innovation	6, 8, 11, 22, 24, 26, 29, 30, 36, 40, 41	27
Description and technical characteristics of technology	Characterization of MD	Assessment of the intrinsic and specific characteristics of MD (e.g., health risk, useful life, contact with the body and exposure time, single or reprocessed use, applicability and hybridization, usability, learning curve, operator-dependent, position occupied in the line of care, technical performance, interoperability and connectivity, infrastructure requirements, infrastructure, and human resources)	5, 6, 8, 9, 11, 11, 12, 16, 17, 20, 22, 22, 23, 24, 24, 25, 26, 26, 27, 27, 29, 29, 30, 30, 31, 32, 33, 34, 34, 35, 36, 37, 38, 38, 39, 40	73
Legal	-	Assessment of ethical and legal requirements. Examples: compliance and alignment with regulatory aspects	1, 3, 8, 7, 10, 11, 13, 14, 16, 17, 18, 22, 23, 24, 25, 26, 27, 28, 29, 30, 31, 32, 33, 34, 35, 36, 39, 40	68
Organizational	Benefit to the healthcare system	Productivity gains, reduction of avoidable waste, reduction of workload, optimization of resources		49
Costs and economic evaluation	Dynamic Pricing	Economic evaluation considering dynamic prices due to gains of scale (productivity, centralization of purchasing), diversity in business models (rental/lending/acquisition regime), marked innovation with constant entrants to the market (market competitiveness)	6, 8, 11, 16, 26, 29, 30, 31, 41	22
	Cost-effectiveness	Measure that compares the cost of a health intervention with its clinical benefits in terms of economic efficiency	2, 12, 18,21, 24, 26, 27, 30, 32, 33	24
	Cost benefit	Total costs of a health intervention with its monetizable benefits	1, 4, 20, 26, 39	12
	Cost-utility	Assessment of the value and impact of a technology, considering factors such as quality of life and patient satisfaction	1, 11, 12, 14, 27	12
	Cost-efficiency	Considers the cost of an intervention per unit of specific clinical outcome	7, 11, 18, 40	10
	Opportunity cost	Value of benefits lost when choosing a health option over other available alternatives	24	2
	Direct and indirect costs	Considers, in addition to acquisition costs, all associated costs during the technological life cycle. Direct and indirect expenses to keep the MD in full and proper functioning (e.g., consumables, accessories and peripherals, installation, training, logistics, supplies, maintenance and technical assistance, disposal and decommissioning)	4, 6, 9, 12, 20	12
	Conditional reimbursement	Payment linked to the generation of more data and evidence that proves the benefits	11, 23, 30, 34	10
	Budget impact	Analysis of the direct costs associated with the adoption of a new technology, considering its impact on available financial resources	11, 14, 20	7
Social participation	Preferences of patients and doctors	Stakeholder involvement. Patient/caregiver involvement strategies and their impact on the decision. Involvement of health professionals. Robust communication and transparency strategies	4, 6, 10, 11, 13, 15, 16, 18, 21, 24, 28, 34, 37	32

Considering the domains of the HTA Core Model^®^ and our collected data, we add “innovation” within the domain of current use. We understand that ethical aspects would already be included in the evidence dimension when they are generated. Table 4 summarizes the current methods used in HTA studies in MD and their connection with some of the domains. The methods employed are not exclusive to any single domain; and can be applied across multiple domains.

**TABLE 4:** Methods associated with domains for evaluating medical devices (Domains and methods of medical device technology evaluation: a systematic review. São Paulo, Brazil, 2024).

Domains^a^	Method/tools/type of analysis	No article Table 2	% citations/total articles
Health problem, current use of technology and innovation	Effect size and generalization: meta regression	34	2
	Outcomes: Multivariate meta-analysis	34	2
Costs and economic evaluation/Health problem, current use of technology and innovation	Bayesian method	11, 23, 30, 34	10
	Multi-Criteria Decision Analysis (MCDA)	11	2
Costs and economic evaluation	Sensibility analysis	4, 6, 11	7
	Consolidated Health Economic Evaluation Reporting Standards (CHEERS)	14, 27, 29	7
	Discrete choice method	11, 15	5
	Markov model	4, 13	5
	Minimum and maximum cost savings values	4, 11	5
	Decision tree	4	2
	RedETS checklist	14	2
	Quality of economic evaluations: Network method	14	2
	Transferability: European Network of Health Economics Evaluation Database	14	2
	Evaluation of comparative effectiveness	30	2
	Probability Bound Analysis (PBA)	32	2
Social participation	EuroQol-5 Dimensions (EQ-5D)	11	2
	Patient Centered Benefit-Risk (PCBR) Framework	28	2

^a^ The domains “Costs and economic assessment” and “Health issue, current use of technology and innovation” from Table 2 were combined in Table 3 due to the shared application of specific methods for their respective purposes.

In the articles analyzed, a wide variety of rearrangements of domains applied or desired in MD HTA were observed depending on the perspective adopted. In general, countries that carry out MD HTA employ similar domains, with differences residing in the healthcare systems’ care models.

The main domains and dimensions for MD HTA are well established, but the limited availability of clinical evidence (41% of the studies) is a significant difficulty. A recent approach to overcoming this gap is the use of Real-World Evidence (RWE) and post-marketing data in countries with robust techno-surveillance systems. This would assist in the transferability of technologies tested in clinical trials to real-life scenarios.

The “description and technical characteristics of technology” domain, represented by the characterization of the MD, received greater emphasis (73% of the studies), highlighting its importance as a starting point for evaluation. The learning curve associated was a significant factor in the score, suggesting a crucial consideration in evaluations for incorporation that is still little explored in terms of demonstrative data.

Legal aspects (68% of the studies) were identified as drivers for establishing quality regulations, but at the same time they were identified as weak in indicating transparent requirements for incorporation.

The organizational domain, which addresses the benefits for the health system, was highlighted in around half of the articles, highlighting, in this analysis, the importance of generating benefits not only for patients, but also for health systems. Although there is a preference for cost-effectiveness assessments (24% of the studies) in the Costs and economic evaluation domain, cost measures are still applied diffusely. However, the budgetary impact, often guided by the cost-effectiveness threshold, is often the decisive factor for incorporating a technology.

With regard to the life cycle dimension, disinvestment emerges as a prominent topic, although still incipient in discussions. Notable innovations include conditional reimbursement (10% of the studies) and consideration of the preferences of both patients and healthcare professionals (32% of the studies).

## Discussion

In our research, we identified domains, dimensions and listed methods that support HTA studies, with a focus on incorporating MD into health systems. By definition, HTA seeks to inform decision-making based on the value of a technology at different points in its life cycle. This value is traditionally supported by the tripod: safety, efficacy and effectiveness. These three aspects were evident in the results of our research. The safety and efficacy domains were referenced with a frequency of 41%, and 44% of the included articles, respectively.

In the costs and economic evaluation domain, cost-effectiveness was the dimension most frequently reported among the included articles (24% of the studies). Legislation is the main driver of good HTA enforcement practices. It was highlighted in 68% of the studies, demonstrating its importance for filling gaps in studies involving MD.

The lack of international standardization of sufficient evidence for market access, less robust regulatory requirements, and the separation of HTA agencies from regulatory structures without alignment between their processes have weakened the conduct of HTA studies to obtain relevant clinical outcomes. Early HTA and initial dialogues involving HTA and regulatory actors to guide study design, sample size, comparator and outcomes selection are essential strategies. Balancing pre-market and post-market evidence, aligning with regulations, pricing policies, reimbursement, and procurement are important to reduce potential redundancies and increase synergy between process [14–17].

In the domain “health problem, current use of technology and innovation,” the evidence dimension (41% of the studies) obtained a similar score to the safety (44% of the studies) and efficacy (41% of the studies) domains. This demonstrates great interest in the topic of producing evidence for MD, considering the difficulties in conducting planned clinical trials, blinding, for example.

Independent initiatives seek alternatives to systematize this information. This is the case of IDEAL Collaboration, an international organization, which is developing an algorithm to guide the collection of pre- and post-clinical RCT data on MD involving complex surgical interventions [18]. The model adds important steps of preclinical and post-market evaluation, as well as indicating when RCTs should be complemented with other types of studies. Additionally, real-world evidence (RWE) studies that adhere to rigorous standards accepted by the scientific community can contribute to MD evidence [19, 20]. However, the transferability of such studies is more difficult to adopt [21, 22]. In light of this fact, the European Network for Health Technology Assessment (EUnetHTA) developed a transferability checklist: EUnetHTA Adaptation Toolkit [22].

Our study demonstrated that the “description and technical characteristics of technology” domain, dimension “characterization of MD,” is the starting point for evaluations, being reported in 73% of the found studies. Overall, studies discuss the need to establish specific methods for conducting HTA in MD. Due to the wide diversity of MD types, a single methodological guideline may not encompass all the specificities and intrinsic characteristics of the plurality of MD. Therefore, studies suggest using clustering criteria through technological characterization (risk class, mode of use, among others) as a strategy to make the process as standardized as possible.

The organizational domain (49% of the studies) was portrayed as as important as the aspect of the “health problem, current use of technology and innovation” domain. The implementation process of MD is also more challenging compared to other technologies, as they commonly require physical, human, and technological infrastructure requirements.

Therefore, the implementation strategy must be clear, robust, and adaptable for the adoption of new technologies in healthcare services. Communication tools are crucial to disseminate information in a diversified and intelligent manner to enable implementation.

Technical and assistance criteria for adoption and coverage should be explicitly stated [23, 24]. Provision for organizational support and stakeholder engagement is necessary. Additionally, establishing tools to monitor and evaluate the results and implementation of these technologies are important [25–27].

Regarding the “health problem, current use of technology and innovation” domain, which initially refers to clinical benefits, the identified gold standard method is the randomized controlled trial (RCT). Conducting this type of study involving MD is more challenging and difficult than with medications. Particularly for blinding and randomization, which may be ethically or technically infeasible.

Furthermore, studies discuss the difficulty of conducting clinical trials due to the characteristics of the MD production sector, predominantly composed of small and medium-sized companies with low expertise in conducting robust randomized clinical trials for MD, scarcity of funding for research, and less commercial reward for investment in R&D. There is a need for a joint effort involving academic, industry, and regulatory experts to assess the feasibility of designing and conducting RCTs [21, 28–32].

Regarding the domains of intrinsic characteristics of MD, iterative HTA approaches throughout the lifecycle of these technologies have been highlighted. The performance of a MD is often dependent on the context of use, infrastructure of the environment where it will be used, operator skill level, lifecycle and durability of components, supply chain and logistics, and interoperability between associated devices [23, 24]. Thus, many factors can influence the extent of benefits, and in the case of diagnostic and MD technologies that are co-dependent on other interventions, evaluating how subsequent patient outcomes are altered is important. These are also relevant points to consider in an economic evaluation.

In turn, the use of MD can impact the health organizational domain in two ways: interaction with the context can hinder its performance and benefits or modify the way services are delivered. To adopt these technologies efficiently, it is important to assess contextualization scenarios. The adoption of MD can be highly dependent on the organizational feasibility of healthcare services. The methodology, especially in economic evaluation studies, should consider intrinsic properties and characteristics specific to the type of MD under evaluation [33]. Iterative economic models can benefit decision-making according to the adopted context. The following tools and models for economic evaluations were mentioned in the reviews: CHEERS [14, 34], similar efficacy comparison, and cost calculation comparison method [35–37]. Basu and Egginton (2019) discuss the importance of documenting the intrinsic properties of MD on the CHEERS checklist to improve the transparency of economic evaluations [33].

There is a significant asymmetry of price information, as MD commonly do not have referenced prices through pricing tables. Dynamic pricing can occur due to factors such as new competitors entering the market, economies of scale and productivity gains, acquisition and contracting modalities, tax and tariff changes in the domestic and international markets, and fiscal benefits granted to institutional profiles. For technologies that require investment and capital expenses, the costs of irrecoverable investments should be weighed, such as the total cost of ownership associated with supply, installation, training, consumables, maintenance, and facilities are important considerations. The different financing models of MD can affect the estimates of economic evaluation studies and budget impact. Conditional coverage, risk-sharing agreements, coverage with evidence development (CED) have been discussed as strategies to minimize uncertainties in clinical and economic evaluation, both due to the learning curve effect, organizational impact, and technological life cycle of MD [18–20, 29, 32, 33, 35, 38–42]. Approaches for the use of multi-criteria decision analysis (MCDA) has also been reported [21, 43–45].

The need to establish criteria for prioritizing HTA demands in MD was addressed in the included studies [18]. The studies presented criteria for prioritization considering relevant aspects, such as: economic, social, organizational, ethical impacts, epidemiological relevance and uncertainty in clinical evidence [15, 46]. Criteria are discussed for various purposes, such as defining and developing risk sharing agreements [39], for divestment [47, 48], as well as criteria for establishing accelerated registries [32].

The main methodological approaches extracted from the included studies are described in a narrative form in the Supplementary Material S5.

Early assessment methods, such as horizon scanning (HS), are essential for an economic sustainability agenda to identify clinically beneficial and cost-effective new technologies, promote their early adoption, and ideally replace less effective or more expensive technologies and practices, as well as contribute to guiding the appropriate timing for conducting assessments [25, 28, 29, 49, 50].

The redundancy and duplication of HTA in MD studies can be exacerbate, especially by the characteristics of rapid development with constant modifications to the product, in addition to the wide possibility of indications for use. Hawlik et al. (2018) discussed the duplicity analysis of HTA MD studies in Europe, highlighting some redundancies, especially for HTA institutes in Spain, where identical technologies were evaluated by up to three different institutes. To avoid redundancy and avoid duplication of efforts, collaborative HTA studies are necessary through interest groups and similar activities that involve the sharing of scientific information [49].

To avoid redundancy and duplication of studies, shared databases and repositories with ongoing and completed studies are relevant. Collaborative activities can occur in the phases of literature search protocols, extraction tables, information on the description and technical characteristics of the technology, as well as in defining the health problem, executive summaries, and full reports. Involvement of stakeholders is necessary to ensure legitimacy, including regulators, payers and policymakers, research and academia, industry, patients and caregivers, healthcare service providers, and social organizations [25, 28, 49, 51, 52]. International collaboration is required to overcome inherent challenges in MD HTA [19].

Fragmentation between decision-making in the national HTA and processes of acquisition and implementation of technologies in local health services is practically inevitable due to the institutional structures. Due to the diversity of configurations available in the MD market, in addition to the specific requirements for local compatibility, installation, training, supply chain and maintenance, the centralized procurement process with a single payer is more challenging and complex when compared to medicines. Thus, after the decision to incorporate a new MD into the health system and for the proper provision of access, under equitable conditions, it is necessary to make efforts to coordinate the entire process to ensure the intended outcomes [15].

Strategies to reduce uncertainties resulting from the fragilities of clinical and economic evidence in decisions on incorporating MD into healthcare systems have been widely discussed. There are variations in definitions, CED, risk-sharing agreements and conditional coverage. Robust databases capable of generating data on the use of these technologies are essential and required [53].

Despite the obtained results, it is important to consider the limitations of this study. The search period was limited to the last 5 years. No documentary survey of methodological guidelines issued by HTA agencies was conducted. Not all actors involved and interested in HTA processes, such as representatives of manufacturers and patients, participated in discussions of research findings.

Recent publications have discussed HTA in MD with specific methodological approaches and objectives. Quintana and Cruz (2022) published the study discussing HTA models focusing on hospital acquisition of MD [54]. Ming et al. (2022) published the study discussing reports issued by HTA Agencies [37]. Freitas et al. (2023) published the study that explores the relevance of distinct value aspects for evaluating different types of medical devices according to stakeholders’ views [55]. Cangelosi (2023) published a systematic literature review to identify relevant studies and criteria used in medical device purchasing or procurement decisions [56].

Our research identified and characterized consolidated and innovative domains and methods to support HTA studies in MD. Through a systematic literature review, our findings can contribute to the field of incorporating MD in health systems.

## Data Availability

Search complete results can be made available upon request to the corresponding author.
